# Role of Noncoding RNAs in the Regulation of P-TEFb Availability and Enzymatic Activity

**DOI:** 10.1155/2014/643805

**Published:** 2014-02-19

**Authors:** Giuliana Napolitano, Luigi Lania, Barbara Majello

**Affiliations:** ^1^Department of Biology, University of Naples Federico II, Naples, Italy; ^2^Department of Molecular Medicine and Medical Biotechnologies, University of Naples Federico II, Italy

## Abstract

P-TEFb is a transcriptional factor that specifically regulates the elongation step of RNA polymerase II-dependent transcription and its activity strictly required for Human Immunodeficiency Virus (HIV) infection and during cardiac differentiation. P-TEFb role has emerged as a crucial regulator of transcription elongation and its activity found finely tuned *in vivo* at transcriptional level as well as posttranscriptionally by dynamic association with different multisubunit molecular particles. Both physiological and pathological cellular signals rapidly converge on P-TEFb regulation by modifying expression and activity of the complex to allow cells to properly respond to different stimuli. In this review we will give a panoramic view on P-TEFb regulation by noncoding RNAs in both physiological and pathological conditions.

## 1. Introduction

The core P-TEFb complex is a hetero-dimer composed of a kinase, (CDK9), and a cyclin subunit of the Cyclin T family (i.e., T1, T2a, and T2b) [1–3].

P-TEFb activity was initially described as essential for transcriptional activation of the Human Immunodeficiency Virus, HIV-1, viral genes as well as for the expression of some cellular genes such as *c-myc*, *hsp70*, and *c-fos* whose transcriptional expression levels are regulated at the elongation phase [4–7]. Genome-wide studies have demonstrated that most of RNAPII-dependent genes are regulated at the elongation step [8–14]. Soon after pre-mRNA transcripts reach the length of about 30 nucleotides, transcription is halted by the negative action of DSIF and NELF complexes [15, 16]. Paused RNAPII is released by the activity of P-TEFb, which phosphorylates the SPT5 subunit of DSIF and the E subunit of NELF as well as the serine residue at position 2 of the RNAPII-Rpb1-CTD (see [15–17] and references therein).

P-TEFb activity is specifically required to allow viral HIV-1 genes to be actively transcribed during infection [2, 6, 18–22]. In addition, it has been shown to be necessary, as part of the p300/GATA4 complex, for transcription of cardiac specific genes such as *Nkx2.5*, *Anf*, and **β*-Myh* [23, 24]. Nevertheless, the list of genes that specifically require P-TEFb activity to be promptly expressed is continuously growing and includes developmental, cellular stress- and cancer-associated genes [25–32].

The P-TEFb role in gene expression is achieved by a fine tuning of its activity in living cells at transcriptional level as well as by its dynamic association with snRNP particles (see [33–35] and references therein). The enzymatic activity of the complex relies on the presence of the 7SK noncoding RNA that binds to Hexim, LARP7, and MePCE and inhibits P-TEFb kinase activity (see [36–39] and references therein). Moreover, recent findings revealed that P-TEFb synthesis is finely regulated by a number of noncoding RNAs (microRNA). Thus, P-TEFb availability and enzymatic activity are largely controlled by several different noncoding RNAs.

## 2. Regulation of P-TEFb Enzymatic Activity by 7SK-Containing snRNP Particles: Dynamic Equilibrium between SC and LC P-TEFb Complexes

In cells, P-TEFb exists in two major forms that are in dynamic equilibrium [31, 37, 40, 41], the core active heterodimer CDK9/Cyclin T (also named small complex, SC) and the inactive 7SK snRNP-bound complex (large complex, LC). In the inactive 7SK snRNP-bound P-TEFb form, the sequestration into the snRNP particle is sufficient to inhibit CDK9 kinase activity. The snRNP contains the noncoding 7SK snRNA and the proteins MePCE (also named BCDIN3), LARP7, and Hexim1 or 2, which can associate as homo- or heterodimers. MePCE and LARP7 are stably bound to 7SK snRNA, while Hexim binding is reversible and is required to inhibit P-TEFb activity. The role of MePCE and LARP7 is to stabilize the integrity of 7SK snRNA as well as the snRNP itself [42–51]. Depending on the cell type, up to 90% of P-TEFb is found in the large inactive complex and the equilibrium between LC and SC determines the overall transcriptional potential activity of the cell. Several different cellular stress signals have been demonstrated to be able to perturb the equilibrium between small active P-TEFb and the 7SK snRNP-bound complex: DNA damage induced by different chemical drugs (camptothecin, doxorubicin, etc.), physical agents (UV light and X-rays), heat, histone deacetylase inhibitors, cardiac hypertrophy, specific intracellular signaling cascades [52–59]. Notably, it has been suggested independently by two research groups that inhibition of transcription itself may determine P-TEFb/7SK snRNP disruption. In the presence of aberrant transcriptional arrest Hexim dissociates from 7SK snRNP and free hnRNPs (viz. hnRNPA1/2, hnRNPQ and hnRNPR) take its place, supporting the notion that the dynamic equilibrium between LC and SC is a mechanism of release of P-TEFb and Hexim from 7SK snRNP [60, 61]. Although precise molecular mechanisms regulating the sequestration/release of P-TEFb from LC remain to be fully elucidated, multiple posttranscriptional modification of 7SK snRNP components are involved as reported elsewhere [32, 62–65].

## 3. miRNAs-Dependent Regulation of P-TEFb Activity in HIV-1 Infection and Latency

Transcription of HIV-1 viral genes requires P-TEFb recruitment on the TAR sequence present on all nascent viral RNAs via direct association between the viral transactivator Tat protein and the Cyclin T1 subunit of the host P-TEFb complex [2, 66–69].

The two major cell types that support productive HIV-1 infection are activated CD4^+^ T lymphocytes and differentiated macrophages, while the main reservoir of HIV-1 latency is represented by resting CD4^+^ T lymphocytes in which P-TEFb activity is under stringent control (see [70] and references therein).

A number of mechanisms have been identified that regulate P-TEFb availability and enzymatic function. Specifically, in CD4^+^ T lymphocytes and macrophages the role of several microRNAs (miRNAs) capable to regulate the expression of the Cyclin T1 subunit has been recently elucidated. In resting CD4^+^ T lymphocytes Cyclin T1 protein levels are very low and dramatically increase upon activation. Similarly, during differentiation of monocytes to macrophages an increase of Cyclin T1 expression is observed. Since levels of Cyclin T1 messenger RNA do not change during CD4^+^ T lymphocytes activation and monocytes differentiation, the increase in Cyclin T1 expression is independent of transcriptional regulation [71–76]. Recent studies provided evidences that Cyclin T1 is under the control of miR-27b, 29b, 150, and 233 in resting CD4^+^ T lymphocytes and miR-198 in monocytes [75]. Abundance of miR-27b, 29b, 150, and 233 decreases upon activation of CD4^+^ T lymphocytes together with an increase of Cyclin T1 protein levels. Interestingly, overexpression of these small noncoding RNAs downregulates Cyclin T1 protein levels in transfected HeLa cells. Besides, it has been shown that in resting CD4^+^ T lymphocytes their inhibition leads to an increase of Cyclin T1 protein levels [75]. In the same study, it has been shown that miR-27b directly binds the Cyclin T1 3′UTR, while no direct interaction has been found for miR-29b, 150, and 233, thus suggesting that in this case the effect on Cyclin T1 expression could likely be indirect.

miR-198 has been shown to target Cyclin T1 3′UTR and inhibit Cyclin T1 expression in monocytes. Ectopic expression of miR-198 in these cells inhibits upregulation of Cyclin T1 protein induced upon differentiation and represses HIV-1 replication and expression of the HIV-1 proviral plasmid in a monocytic cell line. miR-198 is expressed at high levels in primary monocytes and it has been suggested that the refractoriness of these cells to support HIV-1 replication might be due to miR-198-dependent inhibition of P-TEFb via repression of Cyclin T1 expression [74].

Intriguingly, it has been shown that a specific miRNA is produced by the HIV-1 viral TAR element and that this miRNA, localized to the exosomes of infected cells, represses apoptosis by modulating the transcriptional levels of *BIM* and *CDK9* promoters [77, 78].

## 4. miRNAs-Dependent Regulation of P-TEFb in Cardiac Hypertrophy and Cardiac Differentiation

Cardiac hypertrophy is characterized by enlargement of myocytes cell size in response to different stimuli. At molecular level, increase of mRNA synthesis and transcriptional activation of the fetal gene program are at the basis of this cardiac injury [79]. It has been shown that P-TEFb is the limiting factor responsible for a general transcription increase both *in vivo* and *in vitro *[80, 81]. Notably, all hypertrophic stimuli have been shown to lead to release of P-TEFb from its inactive state [79–82]. Although the Jak/STAT pathway has been involved in the release and activation of P-TEFb in the context of cardiac hypertrophy, a miR-1-dependent regulation of CDK9 synthesis during cardiac differentiation and hypertrophy has been recently identified [83].

Similar to cardiac hypertrophy, also normal cardiac development relies on increased cell size, mRNA, and protein synthesis and P-TEFb activity seems to be responsible for these effects. A number of miRNAs have been shown to play a role during cardiogenesis and among them miR-1 has been shown to be involved in P-TEFb regulation [84–90]. In fact, *in vivo* data showed that the 3′UTR of CDK9 messenger RNA is a miR-1 direct target [85].

miR-1 has been previously identified as a muscle-specific miRNA and then found to have a pivotal role in heart development being the earlier miRNA downregulated during cardiac hypertrophy [91, 92].

The presence of miR-1 in ES cells is barely detectable, but upon cardiac differentiation its expression progressively increases with a concomitant reduction of CDK9 expression. These studies suggest that miR-1 regulates myocardial differentiation of ES cells in part by reducing CDK9 availability [85]. Although CDK9 role during cardiac growth is critical, it has been shown that miR-1 targets also Hand2 mRNA during heart development [91]. In line with its role in promoting cardiac differentiation in part reducing the availability of P-TEFb, miR-1 expression is downregulated in cardiac hypertrophy. It has been shown that miR-1 down-regulation is necessary for the upregulation of CDK9, suggesting that the balance between miR-1 and CDK9 (i.e., P-TEFb) plays essential role during cardiac hypertrophy.

It is of note that production of oligoribonucleotides homologous to CDK9 mRNA in miR-1 microinjected one-cell embryo, as well as of miR-1 itself, determines the hyper-activation of CDK9 transcription and the establishment of cardiac hypertrophy in developed mice. For instance, the cardiac injury is inherited in the progeny, due to a phenomenon initially discovered in plant and called “paramutation.” In the case of fertilized eggs, the “paramutation” has been suggested to be due to epigenetic modifications or abnormal forms of CDK9 transcript. Nevertheless, the molecular nature of these heritable alterations has to be clarified [93, 94].

## 5. miRNAs-Dependent Regulation of Cyclin T2 Levels in Leukemia and Spermatogenesis

P-TEFb, as component of super elongation complexes (SECs), has been shown to have a pivotal role in halting hematopoietic differentiation in mixed-lineage leukemia (MLL), a very aggressive subtype of acute myeloid leukemia. SECs are multifactor complexes consisting of members of ELL family proteins, several MLL translocation partners such as members of AFF family proteins, ENL, AF9, and P-TEFb [95–100]. When SECs are aberrantly brought to MLL targets they are able to misregulate *HOX* genes as well as other developmental genes such as Wnt target genes and leukemic stem cell target genes developing MLL leukemias [101–104].

A functional role of miRNAs during hematopoiesis has been highlighted only recently. Interestingly, miR-29a and miR-142-3p have been shown to be severely downregulated in acute myeloid leukemia (AML), a group of blood cancers characterized by the blockage of myeloid differentiation [105–108]. Moreover, data from a recent work demonstrate that miR-29a and miR-142-3p expression levels increase during differentiation of several leukemia cell lines and that their inhibition using specific anti-miRNAs determines a blockage in myeloid differentiation and the consequent development of AML. Furthermore, miR-29a and miR-142-3p are present at lower levels in PBMNCs (peripheral blood mononuclear cells) and BM (bone marrow) of AML patients if compared to normal patients [107, 108]. It has been reported that a key role of miR-29a and miR-142-3p in myeloid differentiation and AML involves regulation of three target genes Cyclin T2 (*CCNT2*), cyclin-dependent kinase 6 (*CDK6*), and TGF-*β* activated kinase 1/MAP3 K7 binding protein 2 (*TAB2*). Notably, while *CDK6* is a target of miR-29a and *TAB2* is a target of miR-142-3p, *CCNT2* is target of both miRNAs. Wang and colleagues showed that Cyclin T2 inhibits myeloid differentiation by increasing their proliferation and that miR-29a and miR-142-3p promote monocytopoiesis in part by regulating Cyclin T2 expression levels. Moreover, abnormal increased levels of Cyclin T2 as well as of *CDK6* and *TAB2* are detected in AML blasts with concomitant reduction of miR-29a and miR-142-3p which further suggests that the two miRNAs regulate myeloid differentiation via these three targets [108].

Cyclin T2 mRNA has also been shown to be target of miR-15a during early spermatogenesis. A microarray study revealed that miR-15a, one of the 28 miRNAs whose expression resulted modified during differentiation, specifically targets 3′UTR of Cyclin T2 mRNA. Down-regulation of miR-15a has been initially related to various cancers as well as to development and differentiation. Data reported from Teng and colleagues showed an inverse relationship between Cyclin T2 expression and miR-15a, suggesting a regulatory loop that is crucial in early spermatogenesis [109].

## 6. Conclusion

Discovery of the noncoding 7SK RNA cellular function in 2001 opened a door to the comprehension of P-TEFb regulation by noncoding RNAs. Since then, a number of studies have clarified the composition and mode of action of the 7SK snRNP regulating P-TEFb equilibrium and activity in the broad spectrum of biological processes in which P-TEFb is involved.

**Figure 1:**
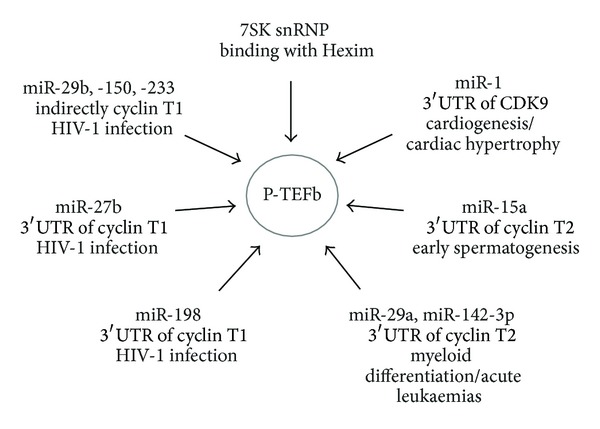
Noncoding RNAs that influence P-TEFb availability and activity are schematically represented. Target 3′UTR and involved biological process are indicated.

More recently, P-TEFb regulation by miRNAs is emerging as schematized in Figure 1. Biology of miRNAs is far to be fully elucidated; nonetheless, despite being considered “tiny players,” they have key roles in a number of developmental and pathological conditions. It is not a surprise that also P-TEFb is an miRNAs target and that several miRNAs are involved in the regulation of P-TEFb availability in different physiologic and pathologic cellular models. It is reasonable to imagine that new players still have to come to light in the near future and that exploring the emerging field of P-TEFb regulation by miRNAs will give new opportunities to shed light on cancer, HIV-1 infection, and the number of pathological conditions in which P-TEFb has a pivotal role.
